# Antegrade balloon dilatation of the duodenal papilla during laparoscopic cholecystectomy versus endoscopic retrograde cholangiography in patients with acute choledocholithiasis: a case control matched study

**DOI:** 10.1007/s00464-024-10909-5

**Published:** 2024-05-29

**Authors:** Severin Gloor, Simone Minder, Bianca Schnell, Gian Andrea Prevost, Reiner Wiest, Daniel Candinas, Beat Schnüriger

**Affiliations:** 1 Department of Visceral Surgery and Medicine, Inselspital, Bern University Hospital, University of Bern, Bern, Switzerland; 2 Department of Surgery, Cantonal Hospital Graubünden, Chur, Switzerland

**Keywords:** Choledocholithiasis, Common bile duct stone, Antegrade balloon dilatation, ERC, Cholecystectomy

## Abstract

**Introduction:**

In acute obstructive common bile duct (CBD) stones endoscopic retrograde cholangiography for CBD stone removal before cholecystectomy (ChE) (‘ERC-first’) is the gold standard of treatment. Intraoperative antegrade balloon dilatation of the duodenal papilla during ChE with flushing of CBD stones to the duodenum (‘ABD-during-ChE’) may be an alternative ‘one-stop-shop’ treatment option. However, a comparison of outcomes of the ‘ABD-during-ChE’ technique and the’ERC-first’ approach has never been performed.

**Methods:**

Retrospective case control matched study of patients suffering from obstructive CBD stones (< 8 mm) without severe pancreatitis or cholangitis that underwent the traditional ‘ERC-first’ approach versus the ‘ABD-during-ChE’ technique. Primary endpoint was the overall Comprehensive Complication Index (CCI®) from diagnosis to complete CBD stone removal and performed ChE.

**Results:**

A total of 70 patients were included (35 patients each in the ‘ERC first’- and ‘ABD-during-ChE’-group). There were no statistical significant differences in terms of demographics and disease specific characteristics between the two study groups. However, there was a not significant difference towards an increased overall CCI® in the ‘ERC-first’ group versus the ‘ABD-during-ChE’ group (14.4 ± 15.4 versus 9.8 ± 11.1, *p* = 0.225). Of note, six major complications (Clavien-Dindo classification ≥ IIIa) occurred in the ‘ERC-first’ group versus two in the ‘ABD-during-ChE’ group (17% versus 6%, *p* = 0.136). In addition, significantly more interventions and a longer overall time from diagnosis to complete clearance of bile ducts and performed ChE was found, when comparing the ‘ERC-first’ group and the ‘ABD-during-ChE’ group (3.7 ± 0.8 versus 1.1 ± 0.4,* p* < 0.001; 160.5 ± 228.6 days versus 12.0 ± 18.0 days, *p* < 0.001).

**Conclusion:**

In patients suffering from acute obstructive CBD stones smaller than 8 mm, compared to the ‘ERC-first’ approach, the ‘ABD-during-ChE’ technique resulted in significantly less interventions and reduced overall treatment time from diagnosis to complete clearance of bile ducts and performed ChE. This comes together with a strong trend of less intervention related complications in the ‘ABD-during-ChE’ group.

Gallstone disease is one of the most prevalent and costly digestive diseases in developed countries. Around 10% of patients with gallbladder stones will be admitted with cholestasis resulting from obstructive common bile duct (CBD) stones [1]. Choledocholithiasis is widespread in the Western population: In Switzerland, 14,689 patients required surgery for cholelithiasis and its complications, and an estimated 20 million Americans suffer from gallbladder disease [2, 3]. An estimated 5–20% of all patients undergoing cholecystectomy also have choledocholithiasis, making this disease a major impact on healthcare systems and the economic burden [4, 5]. Standard treatment of CBD stones in combination with gallbladder stones is endoscopic retrograde cholangiography (ERC) with sphincterotomy and stone removal either before or after prophylactic laparoscopic cholecystectomy (ChE) in a two-step procedure (‘ERC-first’). Of note, stent placing may be required after ERC and sphincterotomy to ensure postinterventional patency of the bile ducts caused by bleeding, swelling of the sphincter region or left behind debris. Those stents have to be removed in a third intervention a few weeks after cholecystectomy.

ERC with sphincterotomy is associated with considerable short and long term morbidity [6–8]. The main complications are post-ERC-pancreatitis (2–5%), impaired sphincter of Oddi function with reflux of duodenal content to the bile ducts and a consecutive higher risk for cholangitis, stone recurrence and even a potential higher risk for cholangiocarcinoma [6–9].

Due to the above-mentioned disadvantages of ERC, surgical bile duct clearance during ChE over a transcystic antegrade approach has been proposed years ago [10]. This anterograde procedure has been described with similar CBD stone clearance rates, less morbidity and a better cost effectiveness compared to the ERC. However, despite being first described more than 20 years ago, providing a low rate of postoperative pancreatitis and a satisfying CBD stone clearance rate, this method has been practiced in specialized centers only [10–14]. Moreover, no comparison to the current gold standard ERC in the treatment of obstructive CBD stones has been published to date.

Recently, in a prospective observational pilot study, a detailed description of the antegrade approach to obstructive CBD stones during laparoscopic ChE has been published from our institution. It has been shown, that the antegrade balloon dilatation (‘ABD-during ChE’) technique is feasible and safe and may offer an alternative to the traditional ‘ERC-first’ approach in patients suffering from obstructive CBD stones of up to 8 mm in size [15]. Therefore, the aim of this study is to compare for the first time the overall morbidity of the traditional ‘ERC-first’ group to the ‘ABD-during-ChE’ group in a retrospective matched case control study. To assess morbidity, the Comprehensive Complication Index (CCI®) was used, in order to include grade and number of complications. Accordingly, the CCI ranges from 0 (uneventful course) to 100 (death) [16].We hypothesize that the overall morbidity, depicted as Comprehensive Complication Index (CCI®) over the entire treatment course from admission until complete CBD stone clearance and laparoscopic ChE will be significantly decreased in the new ‘ABD-during-ChE’ group compared to the traditional ‘ERC-first’ group [16].

## Methods

### Patient inclusion and exclusion criteria

This trial is a monocentric retrospective case control matched analysis of patients diagnosed with acute obstructive CBD stones at the Department of Visceral Surgery and Medicine at Bern University Hospital, University of Bern, Switzerland. The study population comprised of patients with an age ≥ 18 years admitted with obstructive CBD stones ≤ 8 mm and absent severe biliary pancreatitis or cholangitis that underwent either the traditional ‘ERC-first’ or the ‘ABD-during-ChE’ approach. All patients with previous surgical interventions to the CBD or evidence of intrahepatic biliary stones were excluded from analysis.

Choledocholithiasis was suspected in patients with right-sided upper abdominal pain, elevated cholestasis parameters and sonographically visualized cholecystolithiasis or visualized CBD stones on sonography or magnetic resonance cholangiography. The diagnosis of CBD stones was then confirmed either at intraoperative cholangiography (IOC) or ERC.

Severe pancreatitis was defined as pancreatitis with a persistent one-organ dysfunction (> 48 h). Severe cholangitis was defined as cholangitis not responding to initial medical treatment (antibiotics and supportive medication) or with at least one-organ dysfunction. Patients with mild to moderate pancreatitis or cholangitis were included. Mild to moderate pancreatitis was defined as more than threefold elevated serum lipase. Mild to moderate cholangitis was defined according to the Tokyo Guidelines 2018 [17]: The guidelines encompass systemic inflammation (fever, chills, or increased inflammatory markers), cholestasis (jaundice or abnormal liver function tests) and imaging (biliary dilation or evidence of stricture, stone, or stent). Diagnosis of cholangitis was confirmed in case all three parameters have been present.

### Data collection

Data of patients that underwent the ‘ABD-during-ChE’ approach have been collected prospectively from 01/2021 to 04/2022 [15]. The demographics and disease characteristics of the ‘ERC-first’ group were retrospectively collected from the electronic patients’ records. These patients were treated from 01/2019 to 12/2020. Subsequently, the ‘ABD-during-ChE’ patients were matched 1:1 to the ‘ERC-first’ group using sex and age (± 5 years) as matching criteria.

### Primary and secondary endpoints

Primary endpoint was the overall Comprehensive Complication Index CCI® [16]. Of note, the CCI® was calculated from all postinterventional complications occurring during the entire treatment period from diagnosis of CBD stone until complete stone clearance of the biliary tract and performed ChE. The severity of complications was assessed according to the Clavien-Dindo classification of surgical complications or the AGREE classification of adverse events in gastrointestinal endoscopy [18, 19]. Secondary endpoints included the number of interventions, overall hospital length of stay, overall intensive care unit length of stay and the duration of overall treatment from diagnosis of CBD stones until complete stone clearance of the biliary tract and performed ChE. Overall hospital stay was defined as number of nights the patient was hospitalized, accumulated over all interventions.

### Study procedure

#### Antegrade balloon dilatation (‘ABD-during-ChE’) approach

The ‘ABD-during-ChE’ technique has been described in detail previously [15]. Similarly to standard laparoscopic ChE, patients are placed in French- and reverse Trendelenburg position. Over an additional 3 or 5 mm 15 cm port, cholangiography by the insertion of the cholangiography catheter (5 French RX ERCP Cannula Tapered Tip, Boston Scientific, Marlborough, Massachusetts, USA) into the cystic duct is performed. In case of a positive cholangiogram for CBD stones or sludge, a guidewire (Jagwire High performance, 260 cm, 0.035-inch, straight tip, Boston Scientific, Marlborough, Massachusetts, USA) is placed via the cholangiography catheter and forwarded over the duodenal papilla into the duodenum. Over this guidewire, a biliary dilatation balloon (Hurricane™ RX, 6, 8 or 10 mm, 4 cm, Boston Scientific, Marlborough, Massachusetts, USA) is then inserted and advanced to the papillary level. The size of the balloon is adjusted to the size of the stone and was previously estimated comparing the diameter of the inserted 5 mm grasper under fluoroscopy. After checking the position of the balloon under fluoroscopy, sphincteroplasty is executed for two minutes with 10 atmospheres (atm) pressure. Afterwards, the guidewire as well as the biliary dilatation ballon is removed and the cholangiography catheter reinserted again. As a final step, the CBD is flushed with 20-40 ml of saline and the final result is controlled by cholangiography before ChE is finalized. If persisting stones were detected after flushing, stones are pushed to the duodenum by the gently inflated dilation balloon along the reinserted guidewire.

In case, CBD stone clearance could not be achieved by the above described ‘ABD-during-ChE’ technique, ChE was finalized and an ERC was subsequently performed at the same hospital stay.

#### Endoscopic retrograde cholangiography (‘ERC-first’) approach

ERC is a minimally invasive endoscopic procedure to diagnose and remove CBD stones. A flexible endoscope is inserted through the patient's mouth, down the esophagus, and into the stomach and duodenum. After visualization of the ampulla of Vater, a catheter (sphincterotome) is inserted through the endoscope and into the bile duct. For cholangiography, a contrast dye is injected into the CBD and fluoroscopy is used to identify the location and size of CBD stones. A guidewire is advanced through the cannula and into the CBD and a sphincterotomy is eventually performed. Over the guidewire, a balloon catheter is introduced above the level of the stone. The balloon is then carefully inflated and gently pulled back into the duodenum until CBD stone extraction has been achieved. Fluoroscopy is performed to confirm that all CBD stones have been removed. Dilatation of the papillary orifice may have to be performed in order to facilitate extraction of bigger stones. Sometimes, biliary stents have to be placed in order to maintain patency of the biliary tract. Finally, the cannula and endoscope are removed, and the patient is monitored as they recover from sedation.

Few days to weeks after the ERC, laparoscopic ChE is performed in a second intervention to remove the main source of gallstones in order to prevent from further biliary complications. In case a CBD stent is inserted at the initial ERC, an additional ERC (third intervention) for removal of the stent has to be performed after ChE.

### Statistical analyses

Quantitative and qualitative variables are expressed as mean (standard deviation) or frequency (percentage). The matching process of the study groups by case control matching was performed by SPSS® version 25 (IBM, Armonk, New York, USA). Matching criteria included sex and age (± 5 years). Demographics, disease characteristics as well as primary and secondary endpoints were compared using Fisher exact test for categorical variables and the Mann–Whitney U test for continuous variables, as appropriate. P < 0.05 was considered statistically significant. Statistical analyses were performed using SPSS® version 25 (IBM, Armonk, New York, USA).

## Results

**Fig. 1:**
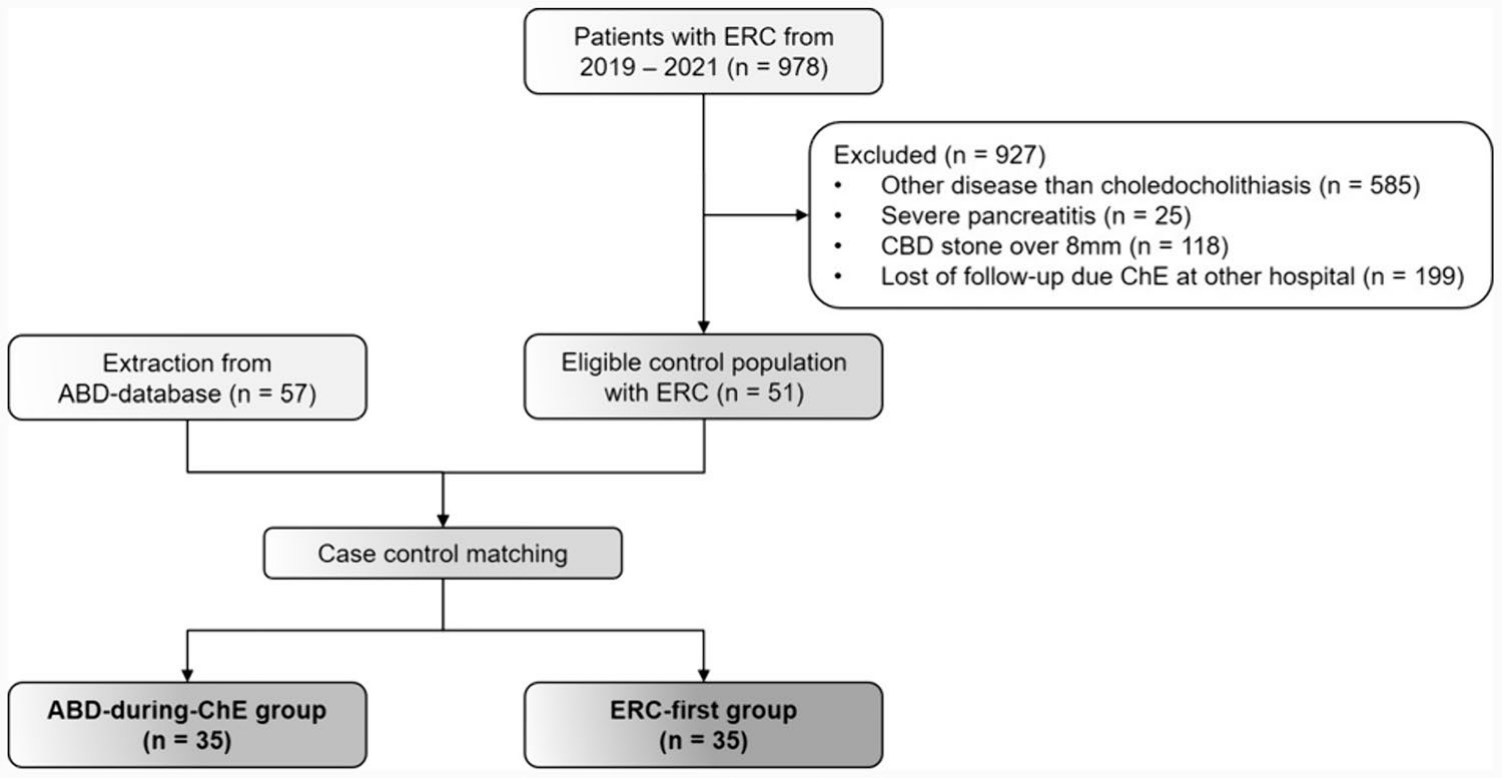
Study outline of the two study groups

Figure 1 shows the study outline. The ‘ABD-during-ChE’ group consisted of 57 patients from the previous prospective observational pilot study [15]. These patients were 1:1 matched to the ‘ERC-first’ group. The ‘ERC-first’ group was recruited from a total of 978 patients who underwent an ERC between 01/2019 and 12/2021 at Bern University Hospital. After excluding patients that underwent ERC for other reasons than CBD stones, patients with a stone size of > 8 mm, patients with severe pancreatitis/cholangitis, and 199 patients who underwent cholecystectomy at another hospital, a total of 51 eligible ‘ERC-first’ patients remained for further analysis.

After above-mentioned patient recruitment process, a total of 108 patients, who underwent treatment for CBD stones < 8 mm by the ‘ABD-during-ChE’ or the ‘ERC-first’ approach and absent severe pancreatitis or cholangitis were available for case control matching (Fig. 1). After case control matching a total of 70 patients (35 patients in the ‘ABD-during-ChE’ group and 35 patients in the ‘ERC-first’ group) were available for further analyses regarding primary and secondary endpoints.

### Demographics

Demographics and disease specific data of the study populations are delignated in Table 1. After matching, the ‘ABD-during-ChE’ group was similar to the ‘ERC-first’ group with regards to age, sex, body mass index, CRP level on admission, ASA classification and the Charlson-Comorbity index, respectively. Moreover, due to the inclusion/exclusion criteria, there were no severe pancreatitis or cholangitis or CBD stones > 8 mm in both groups.

**Table 1:** Demographics and disease specific variables of patients that underwent antegrade balloon dilatation (ABD) or endoscopic retrograde cholangiography (ERC) due to choledocholithiasis

Variable	Total (*n* = 70)	ABD-during-ChE group (*n* = 35)	ERC-first group (*n* = 35)	*p-*value*
Female sex, *n* (%)	38 (54)	19 (54)	19 (54)	*1.000*
Age (years), mean ± SD	61.6 ± 18.3	61.3 ± 18.3	61.8 ± 18.6	*0.883*
Body mass index (kg/m^2^), mean ± SD	28.6 ± 6.0	27.7 ± 5.5	29.4 ± 6.4	*0.247*
Presence of gastrojejunostomy, *n* (%)	4 (6)	3 (9)	1 (3)	*0.307*
High ASA-classification, *n* (%)	32 (46)	15 (43)	17 (49)	*0.634*
1	4 (6)	3 (9)	1 (3)	*0.307*
2	34 (49)	17 (48)	17 (49)	*1.000*
3	27 (38)	12 (34)	15 (43)	*0.645*
4	5 (7)	3 (9)	2 (5)	*0.609*
Present mild/moderate cholangitis at diagnosis, *n* (%)	26 (37)	13 (37)	13 (37)	*1.000*
Present mild/moderate pancreatitis at diagnosis, *n* (%)	14 (20)	10 (29)	4 (11)	*0.075*
C-reactive protein at diagnosis (mg/L), mean ± SD	51.3 ± 70.1	59.6 ± 72.0	42.8 ± 68.2	*0.213*
Serum bilirubine at diagnosis (mg/dL), mean ± SD	55.3 ± 60.6	38.7 ± 34.3	71.9 ± 75.6	*0.091*
Serum lipase at diagnosis (U/L), mean ± SD	773.2 ± 2050.7	992.3 ± 2156.9	547.5 ± 1942.2	*0.093*
Charlson-Comorbidity Index, mean ± SD	3.5 ± 3.2	3.4 ± 3.2	3.7 ± 3.2	*0.634*

*ASA* American Society of Anesthesiology, *SD* standard deviation

*Mann–Whitney U (numerical variables) or Fisher exact test (categorical variables)

However, there was a trend towards more mild to moderate pancreatitis and higher serum lipase levels on admission in the ‘ABD-during-ChE’ compared to the ‘ERC-first’ group (29% versus 11%, *p* = 0.075; 992 U/L versus 548 U/L, *p* = 0.093). In contrast, bilirubin levels tended to be higher in the ‘ERC-first’ compared to the ‘ABD-during-ChE’ group (72 mg/dL versus 39 mg/dL, *p* = 0.091) (Table 1).

### Primary endpoint

Table 2 summarizes overall CCI® of the two study groups. The ‘ABD-during-ChE’ group showed a lower overall CCI® compared to the ‘ERC-first’ group (‘ABD-during-ChE’ group 9.8 versus ‘ERC-first’ group 14.4, *p* = 0.225), however this difference was not statistically significant.

**Table 2:** Primary and secondary endpoints of the ‘ABD-during-ChE’ group versus ‘ERC-first’ group

Variable	Total (*n* = 70)	ABD-during-ChE group (*n* = 35)	ERC-first group (*n* = 35)	*p*-value**
Conversion to open ChE, *n* (%)	5 (7)	1 (3)	4 (11)	*0.167*
Duration of ChE (minutes), mean ± SD	99.0 ± 30.5	110.7 ± 26.2	84.3 ± 29.6	< *0.001*
Patients with at least one complication, *n* (%)	42 (60)	19 (54)	23 (66)	*0.333*
Highest complication grade per patient				
Complication grade I, *n* (%)	22 (31)	12 (34)	10 (29)	*0.609*
Complication grade II, *n* (%)	12 (17)	5 (14)	7 (20)	*0.529*
Complication grade IIIa, *n* (%)	6 (9)	2 (6)	4 (11)	*0.397*
Complication grade IIIb, *n* (%)	0	0	0	
Complication grade IVa, *n* (%)	2 (3)	0	2 (6)	*0.154*
Complication grade IVb, *n* (%)	0	0	0	
Complication grade V, *n* (%)	0	0	0	
Patients with at least one major complication (CD ≥ IIIa), *n* (%)	8 (11)	2 (6)	6 (17)	*0.136*
Overall CCI®, mean ± SD	12.0 ± 13.5	9.8 ± 11.1	14.4 ± 15.4	*0.225*
Cumulative CCI® from ERC, mean ± SD	3.3 ± 9.1	0	6.5 ± 12.1	< *0.001*
CCI® from ChE, mean ± SD	9.5 ± 12.0	9.5 ± 11.0	9.4 ± 13.0	*0.690*
Overall hospital length of stay (days), mean ± SD	7.2 ± 1.4	5.0 ± 3.0	9.3 ± 5.4	< *0.001*
Overall intensive care length of stay (days), mean ± SD	0.1 ± 0.5	0	0.1 ± 0.7	*0.317*
Number of interventions*, mean ± SD	2.4 ± 1.4	1.1 ± 0.4	3.7 ± 0.8	< *0.001*
Time from diagnosis to end of treatment (days), mean ± SD	86.3 ± 177.5	12.0 ± 18.0	160.5 ± 228.6	< *0.001*
90-day mortality, *n* (%)	0	0	0	

*CCI* Comprehensive Complication Index, *CD* Clavien–Dindo score, *ChE* cholecystectomy, *SD* standard deviation

*From diagnosis of choledocholithiasis until central bile duct stone clearance, removal of stents and ChE

**Mann–Whitney *U* (numerical variables) or Fisher exact test (categorical variables)

A trend towards more major complications (Clavien-Dindo classification ≥ IIIa) were found for the ‘ERC-first’ versus the ‘ABD-during-ChE’ group (17% versus 6%, *p* = 0.136). In the ‘ERC-first’ group 8 major complications occurred in 6 different patients including post-ERC cholangitis with need of ICU care (*n* = 1), post-ChE cholangitis with need of ICU care (*n* = 1), unplanned re-ERC after unsuccessful ERC (*n* = 3), unplanned ERC due to a CBD stent dysfunction (*n* = 1), and post-ChE fluid collection with transcutaneous drain insertion (*n* = 2). In contrast, in the ‘ABD-during-ChE’ group two patients suffered from two major complications. One patient underwent ERC due to increasing cholestasis after ChE and the other patient underwent unplanned negative gastroscopy during follow-up due to inconclusive upper gastrointestinal symptoms.

A total of 84 minor complications (Clavien-Dindo ≤ II) occurred in both study groups. In the ‘ABD-during-ChE’ group overall 36 minor complications occurred in 19 patients and in the ‘ERC-first’ group 48 minor complications occurred in 23 patients. With 37% of all patients (*n* = 26), constipation, defined as the need of laxative medication and without the need of gastric tube insertion, was in both study groups by far the most frequent minor complication.

Specific CCI® resulting from the ERCs or ChE are delineated and compared between the two study groups (Table 2). The CCI® resulting from the ERC specifically was significantly higher in the ‘ERC-first’ group compared to the ‘ABD-during-ChE’ group (‘ERC-first’ group 6.5 versus ‘ABD-during-ChE’ group 0.0, *p* < 0.001). The most common ERC-related complications included postinterventional cholangitis in 7% (*n* = 5), post-ERC pancreatitis in 4% (*n* = 3) or postinterventional anemia in 4% (*n* = 3). In the ‘ABD-during-ChE’ group there was no occurrence of postoperative cholangitis or pancreatitis. During planned laparoscopic cholecystectomy, one patient in the ‘ABD-during-ChE’ group needed a conversion to an open cholecystectomy compared to 4 patients in the ‘ERC-first’ group (3% versus 11%, *p* = *0.167*).

The CCI® resulting from the ChE solely were similar between the two study groups (‘ERC-first’ group 9.4 versus ‘ABD-during-ChE’ group 9.5, *p* = 0.690). The ChE related complications included postoperative paralytic ileus in 4% (*n* = 3), postoperative intraabdominal fluid collection in 3% (*n* = 2) or superficial surgical site infection in 1% (*n* = 1).

### Secondary endpoints

**Fig. 2:**
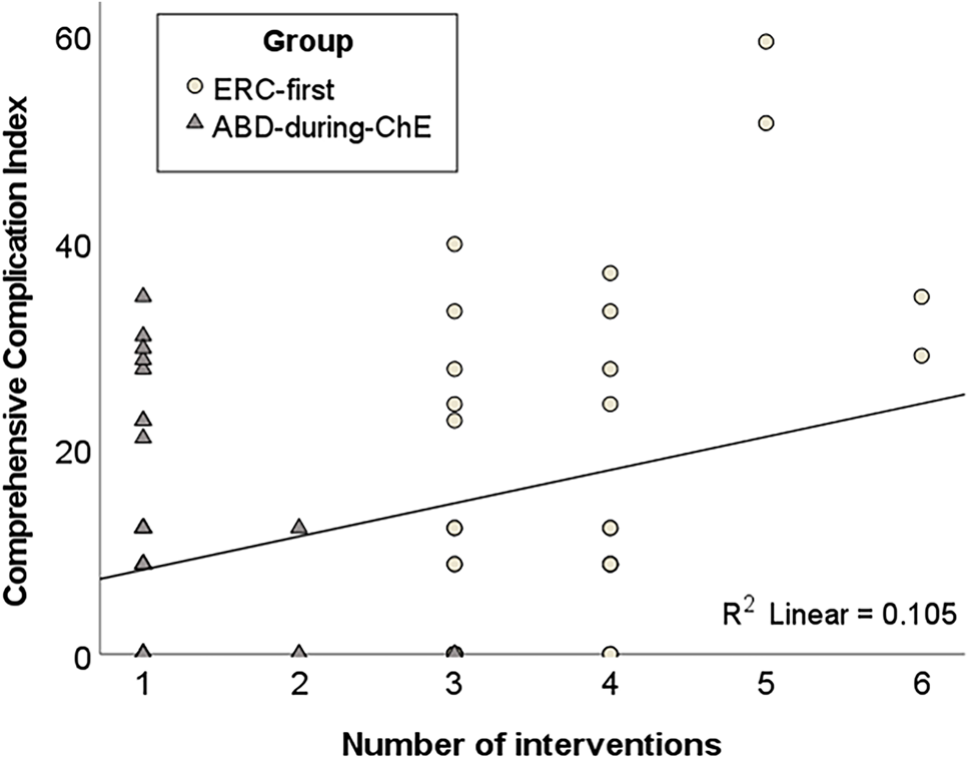
Correlation between the number of interventions during the entire treatment period and the Comprehensive Complication Index

In the ‘ABD-during-ChE’ group a mean number of 1.1 ± 0.4 interventions were needed until finalization of treatment, whereas in the ‘ERC-first’ group 3.7 ± 0.8 interventions were required (*p* < 0.001). Ultimately, the CCI® increased with increasing number of interventions (Fig. 2).

The CBD stone clearance rate or successful antegrade balloon dilatation in the ‘ABD-during-ChE’ was 94% (33 of 35 patients). The two patients with unsuccessful ‘ABD-during-ChE’ approach were a 56-year old female patient that underwent ‘ABD-during-ChE’ due to acute choledocholithiasis with concomitant mild pancreatitis and a 39-year old male patient with acute choledocholithiasis. In both patients the intubation of the cystic duct with the cholangiography catheter or the guidewire intraoperatively was not achievable. Therefore these two patients underwent ERC at the first day after operative treatment with extraction of sludge from the CBD. Further follow-up of these two patients was uneventful.

Similarly, hospital length of stay as well as the overall time from diagnosis to complete clearance of bile ducts and performed ChE was significantly longer for the ‘ERC-first’ group versus the ‘ABD-during-ChE’ group (160.5 ± 228.6 days versus 12.0 ± 18.0 days, *p* < 0.001) (Table 2). In contrast, due to the intraoperative clearance of bile ducts and balloon dilatation of the Sphincter of oddi at ChE, the duration of the ChE increased significantly in the ‘ABD-during-ChE’ group versus the ‘ERC-first’ group (110.7 ± 26.2 minutes versus 84.3 ± 29.6 minutes, *p* < 0.001).

## Discussion

The current study shows that in patients suffering from obstructive CBD stones < 8 mm, the ‘ABD-during-ChE’ approach has a very high rate (94%) of CBD stone clearance in combination with a low total number of interventions. This stands in contrast with the ‘ERC-first’ approach, where all patients required at least two interventions to treat CBD stones including the prophylactic ChE. Moreover, treatment with the ‘ABD-during-ChE’ approach had a lower rate of clinically and outcome-relevant complications classified above Clavien-Dindo grade 3a, compared to the ‘ERC-first’ group. This indicates that ‘ABD-during-ChE’ should be considered as an efficient treatment alternative to ‘ERC-first’ in patients suffering from obstructive CBD stones < 8 mm. Especially in times of progressing shortage of hospitals’ personnel and infrastructural resources, the ‘ABD-during-ChE’ may be a valuable and lean alternative to the traditional two-step ‘ERC-first’ approach.

The disease of choledocholithiasis is common in western population. A total of 14′689 patients required ChE due to cholelithiasis and its complications in 2021 in Switzerland and estimated 20 million Americans suffering from gallbladder disease [2, 3]. Estimating that 5% to 20% of all patients undergoing cholecystectomy have simultaneous choledocholithiasis [4, 5], the disease has a very high impact on health care systems resulting in a significant economic burden. The ‘ABD-during-ChE’ technique offers a lean management for this group of patients with a significantly reduced total number of interventions and with it, according the current study, a significantly reduced treatment period by more than 5 months compared to the traditional ‘ERC-first’ approach. The five-month time from diagnosis to the end of treatment in the ‘ERC-first’ group is the result of a generous use of initial CBD stent insertion. It is common practice at our institution to achieve immediate biliary drainage by inserting a CBD stent in patients with acute obstructive CBD stones. This shortens the duration of the initial emergency ERC, which is favorable in terms of utilization of personnel resources and potentially reduces interventional morbidity. Secondary elective ERC for complete CBD clearance and stent removal is particularly advantageous in complex CBD stone situations. The rather high time from diagnosis to the end of treatment in the ‘ABD-during-ChE’ group is due to four outlying patients. These patients had a mean hospital length of stay of 5.8 days (± 1.7). The time from diagnosis to the end of treatment was prolonged as two patients received endoscopy due to persistent symptoms, one patient needed re-hospitalization due to cholangitis and one patient underwent CBD stent removal.

After elimination of non-clinically and outcome-relevant minor complications (Clavien-Dindo grades I and II), the morbidity of the ‘ABD-during-ChE’ was low with 6% major complications compared to 17% major complications in the ‘ERC-first’ group. This percentage was as low as reported in other studies investigating ‘ABD-during-ChE’ [11, 13, 14], where no major complications were described. Moreover, in the current study, ERC was comparable to other studies regarding major complications [20, 21]. Noticeable is the high percentage of overall complications according to Clavien-Dindo in the current study. This may be explained by the meticulous documentation even of minor complications (e.g., constipation, anemia, or superficial surgical site infection). Whereas other studies assessed major complications only, the current trial used the CCI® as an instrument which integrates also minor complications.

The higher CCI® in the ‘ERC-first’ group may also be explained by the increased number of interventions compared to the ‘ABD-during-ChE’ group (Fig. 2). In the current study, the one-stop-shop-character of the ‘ABD-during-ChE’ approach proved to reduce the number of interventions by three. It is important to realize, that every intervention carries its own risk in terms of complications, which is why the indication for every intervention should be carefully examined. This includes both surgical and anesthesiological risks. Of note, according the literature, significant unplanned events are occurring in up to 23% of all patients undergoing ERC [22, 23].

The surgical treatment of acute obstructive CBD stones during laparoscopic cholecystectomy is mentioned in the guidelines for endoscopic management of CBD stones of the European Society of Gastrointestinal Endoscopy (ESGE) and the American Society of Gastrointestinal Endoscopy (ASGE) as well, but with weak recommendation as existing literature is of moderate quality [24, 25]. The current study is the first that compares the ‘ABD-during-ChE’ to the ‘ERC-first’ approach, which is regarded the gold standard in the treatment of acute choledocholithiasis. The ‘ABD-during-ChE’ approach is one of several possible intraoperative approaches to the CBD (e.g. laparoscopic choledochotomy, transcystic choledochoscopy and removal of CBD stones with a Dormia basket). However, no comparative studies to the current ‘ABD-during-ChE’ approach exist. Often studies examined a combination of different intraoperative approaches on limited numbers of patients, making it difficult to draw any conclusions [26–28]. Moreover, surgical techniques requiring choledochotomy result in more postoperative morbidity due to its invasiveness [29, 30]. In contrast, the ‘ABD-during-ChE’ approach provides a high CBD stone clearance rates, low morbidity and can be carried out with relatively little technical effort.

Even though the current study has clear limitations due to the retrospective design, the limited number of patients, a potential selection bias towards less complicated patients treated by ‘ABD-during-ChE’ and a limited follow-up, the use of stringent inclusion criteria and the case control matching have resulted in two comparable groups. Moreover, the size of differences in the number of interventions observed are partially related to the local clinical practice of liberal CBD stent placement at initial ERC (‘ERC-first’ group) and therefore, generalizability to other institutions is limited. Validation of these results in a randomized controlled setting is warranted in order to generalize indication and effectiveness for ‘ABD-during-ChE’ in acute choledocholithiasis and to strengthen evidence.

## Conclusion

For treatment of acute obstructive CBD stones in uncomplicated patients with smaller CBD stones than 8 mm, the’ABD-during-ChE’ approach resulted in significantly less overall interventions and a trend towards less intervention related morbidity compared to the’ERC-first’ approach. Moreover, there is a great benefit regarding reduced overall time from diagnosis to finalization of treatment including CBD stone clearance and ChE. To generalize these results and to improve the scientific evidence, prospective randomized controlled trials are needed.

## Abbreviations

ABD: Antegrade balloon dilatation
ABD-during-ChE: Antegrade balloon dilatation during cholecystectomy
ASGE: American Society of Gastrointestinal Endoscopy
CBD: Common bile duct
CCI®: Comprehensive Complication Index
ChE: Cholecystectomy
ERC: Endoscopic retrograde cholangiography
ESGE: European Society of Gastrointestinal Endoscopy
IOC: Intraoperative cholangiography
